# Impact of respiratory motion on dose to the airways in central and ultra-central lung stereotactic ablative body radiotherapy

**DOI:** 10.1016/j.phro.2026.100937

**Published:** 2026-02-26

**Authors:** Evan Keane, Gerard G Hanna, Serena O’Keefe, Pierre Thirion, Ciaran Malone

**Affiliations:** a Centre for Physics in Health and Medicine, UCD School of Physics, University College Dublin, Dublin, Ireland; b St. Luke’s Radiation Oncology Network, Dublin, Ireland; c Applied Radiation Therapy Trinity, Discipline of Radiation Therapy & Trinity St. James’s Cancer Institute, Trinity College Dublin, Dublin, Ireland; d School of Medicine, Trinity College Dublin, Dublin, Ireland; e Erasmus MC Cancer Institute, University Medical Center Rotterdam, Department of Radiotherapy, Netherlands

**Keywords:** Stereotactic ablative body radiotherapy, Deformable image registration, Airways, Dose accumulation, Planning risk volume

## Abstract

**Background and purpose:**

Stereotactic ablative body radiotherapy (SABR) achieves excellent local control in medically inoperable non-small cell lung cancer. However, treating central and ultra-central tumours carries significant risks to adjacent critical structures, notably the central airways. This study evaluated the suitability of average value intensity projection (AvIP) imaging for airway delineation and assessed the adequacy of a 5 mm planning risk volume (PRV), by quantifying the influence of respiratory motion on dose to the airways using four-dimensional (4D) dose accumulation.

**Materials and methods:**

Eighteen patients (11 ultra-central, 7 central) treated with 60 Gy in 8 fractions were retrospectively analysed. Clinical 4D computed tomography (CT) datasets were used to capture 10 respiratory phases. Central airway contours (trachea, main bronchi, bronchial tree) were delineated on the AvIP and deformably propagated across all phases. Dose was recalculated per phase and accumulated back into a single distribution using the deformable registration for comparison with the AvIP-based plan.

**Results:**

Maximum differences of 5 Gy were observed for the airway *D_0.1 cm3_*, with an average absolute difference of 0*.*95 *±* 1*.*7 Gy. Notably, 28% of patients had differences >2 Gy, and 17% exceeded 3 Gy. The greatest dose discrepancies occurred in cases with tumours inferior to the first-order bronchi and with breathing motion >6 mm. Five patients’ plans exceeded dose–volume constraints when respiratory motion was accounted for.

**Conclusions:**

Dose to the airways can be significantly underestimated when motion is not incorporated into treatment planning. A uniform 5 mm PRV may be inadequate in some central/ultra-central SABR cases. Patient-specific internal risk volumes merit investigation.

## Highlights

• Accounting for respiratory motion caused airway *D_0.1 cm3_* increases of up to 5 Gy.
• *D_0.1 cm3_* differences exceeded 2 Gy in 28% of the study cohort.
• Tumours moving over 6 mm showed the largest dose discrepancies.
• Standard planning approaches masked dose violations in 5 of 18 patients.

## Graphical abstract

**Figure:**
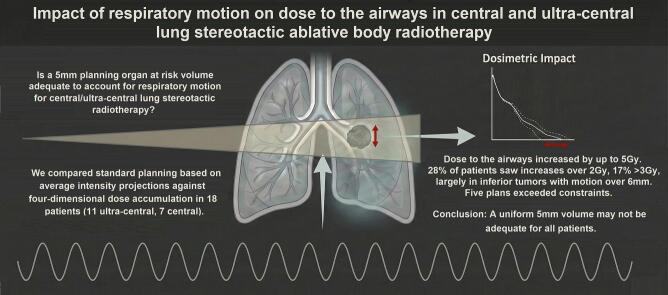

## 1 Introduction

Lung cancer is the leading cause of cancer-related death worldwide, accounting for one in five cancer related deaths, with non-small cell lung cancer (NSCLC) representing approximately 85% of new diagnoses [1]. A significant portion of patients with early-stage NSCLC are deemed medically inoperable due to advanced age, chronic obstructive pulmonary disease (COPD), or cardiovascular comorbidity [2]. For these patients, stereotactic ablative body radiotherapy (SABR) has emerged as the standard of care, offering local-control rates approaching those of surgical resection while maintaining a non-invasive outpatient workflow [3], [4], [5], [6], [7].

The dosimetric benefit of SABR, which involves delivering very high fractional doses with steep dose gradients, relies on geometric certainty. While this is readily achieved for peripheral tumours, centrally or ultra-centrally located lesions (i.e., within 2 cm of the proximal bronchial tree) lie adjacent to critical serial organs, where exceeding dose tolerances can result in severe toxicities [8], [9]. In particular, small hotspots in the trachea and main bronchi can lead to catastrophic bronchopulmonary haemorrhage [10]. Consequently, planners adopt conservative dose-volume constraints (DVCs) and routinely expand the airway contour by 5 mm to create a planning risk volume (PRV) [11]. This empirical margin accounts for setup uncertainty and intra-fraction drift, but neglects individualised respiratory motion.

Respiration displaces the airway by several millimetres in all dimensions, a magnitude comparable to the entire PRV expansion [12]. SABR planning typically delineates organs at risk (OARs) on the average-intensity projection (AvIP), or on a single phase of four-dimensional CT (4DCT). However, AvIP suppresses phase-specific extremes, potentially masking the transient proximity of the airway to high-dose voxels.

Deformable image registration (DIR) enables estimation of the delivered 4D dose by deforming each respiratory phase back to the reference geometry and accumulating energy deposition across time [13]. Prior studies using DIR in liver and heart have revealed clinically meaningful deviations from static plans [14], [15], [16]. However, to our knowledge, no systematic evaluation has been performed on the central airways in lung SABR. Given the severe toxicity profile observed in trials such as HILUS (grade 5 events in approximately 13%) [10], underestimation of dose to the airways may have serious clinical implications.

Therefore, this study investigated whether AvIP-based planning accurately predicts maximum dose to the airways or whether deformable image registration (DIR)–based 4D dose accumulation reveals systematic under- or over-estimation. The influence of breathing-induced airway motion on these discrepancies was evaluated, along with the adequacy of a conventional 5 mm planning risk volume (PRV). In addition, patient- and tumour-related factors, particularly cranio-caudal tumour position, were examined for their ability to predict clinically significant deviations (>2 Gy) from the planned dose to the airways.

## 2 Materials and methods

### 2.1 Patient cohort

Eighteen medically inoperable patients treated between January 2022 and March 2024 for a single centrally located lung lesion were consecutively identified from the departmental SABR database and the Stereotactic Ablative Radiation Therapy Of UltRaCEntral (SOURCE) trial (ClinicalTrials.gov ID NCT04375904) screening log. Centrally located disease was defined geometrically as any gross tumour volume (GTV) situated within 2 cm of the proximal bronchial tree or abutting the mediastinal or pericardial pleura. Tumours impinging on the spinal canal or brachial plexus were also categorised as central. All patients received 60 Gy in eight fractions using volumetric-modulated arc therapy (VMAT). Eleven lesions fulfilled the SOURCE definition of “ultra-central,” while the remaining seven were classified as “central”.

### 2.2 Simulation and image acquisition

Patients were positioned supine on a custom three-quarter length vacuum cushion with both arms raised. Respiratory motion was assessed in the CT-simulation suite using the Real-time Position Management (RPM) system (Varian Medical Systems, California, USA). Only patients with a stable, near-sinusoidal breathing trace were accepted for SABR. A four-dimensional CT (4DCT) was acquired with phase binning on a Discovery CT-590RT scanner (GE Healthcare, Wisconsin, USA), with an RPM marker block placed immediately inferior to the xiphisternum. All patients were treated under a “free breathing with backup gating” protocol. The gating threshold was calculated using Eq. (1) from the amplitude of the patient’s breathing trace and the maximum tumour motion measured on the 4DCT.

(1) $$\mathrm{Gating}\;\mathrm{threshold}=\left(\frac{\mathrm{Breathing}\;\mathrm{trace}\;\mathrm{amplitude}}{\mathrm{Max}\;\mathrm{tumour}\;\mathrm{amplitude}}\right)*5mm$$

The applied margin was limited to a maximum of 5 mm. For calculated margins below this limit, the exact value (rounded to 0.5 mm) was used. Average-intensity projection (AvIP) and maximum-intensity projection (MIP) datasets were automatically generated and exported, along with the individual phase volumes, to the Eclipse treatment-planning system (Varian Medical Systems, California, USA).

### 2.3 Target and organ at risk delineation

All contours were created on the AvIP CT in Eclipse v15.6 (Varian Medical Systems, California, USA). The iGTV was outlined by a thoracic radiation oncologist on the AvIP, using the MIP and the end-inhale/end-exhale phase images as guidance. The iGTV delineation was also reviewed and edited on all phases as standard. A uniform 5 mm internal target volume expansion was applied to the iGTV to generate the planning target volume (PTV). OARs were delineated on the AvIP by trained radiation therapists and verified by the same oncologist. The airway was segmented into trachea and first-order bronchi, which were combined into a single “Airway” structure. Each organ at risk (OAR) received a 5 mm planning risk volume (PRV) unless clinical constraints (Supplementary Tables 2 and 3) necessitated a 3 mm margin. Only 5 mm PRVs were analysed in this study.

### 2.4 Airway and breathing motion assessment

Breathing amplitude was recorded by CT radiation therapists from the 4DCT respiratory trace during simulation and documented in the institutional 4DCT evaluation form. The trace amplitude, corresponding to peak-to-trough displacement of the external surrogate marker, was extracted from this form and entered into an anonymised database for analysis.

Airway motion was quantified retrospectively in Eclipse. The end-inhale and end-exhale respiratory phases were identified from the 4DCT evaluation form and loaded alongside the treatment plan. Airway contours were overlaid on both datasets in lung windows. Using the “Measure Distance” tool, the displacement of the airway point nearest the PTV was measured between corresponding positions on the end-inhale and end-exhale phase images. This superior–inferior excursion was recorded to the nearest millimetre and used as the estimate of patient-specific airway motion.

### 2.5 Plan optimisation

VMAT plans were optimised on the AvIP using two coplanar 200° arcs of 6 MV photons; three non-coplanar arcs were used when needed to meet OAR constraints (Supplementary Table 2). Dose was calculated with Acuros XB v15.6 (Varian Medical Systems, California, USA) (dose-to-medium, 1 mm grid).

### 2.6 Deformable image registration and dose accumulation

**Fig. 1:**
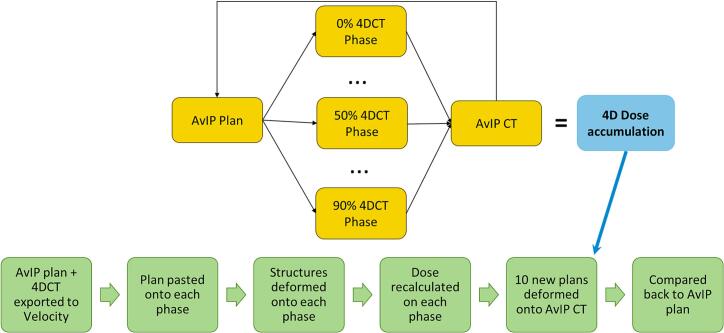
Deformable image registration (DIR) using Velocity AI software was employed to propagate airway contours from the original treatment plan, based on the AvIP, onto each phase of the 4DCT. This method allowed accurate alignment of anatomical structures across respiratory phases, essential for precise dose calculation and accumulation. Dose calculations were performed individually for each respiratory phase, and the DIR was again utilised to accumulate these doses into a single representative dose distribution.

All CT volumes and the reference radiotherapy plan were exported to Velocity AI v4.1 (Varian Medical Systems, California, USA). The “Plan Generation-Actor” workflow performed a deformable multi-pass registration between the AvIP and each 4DCT phase, automatically propagating selected structures (airway, airway +5 mm, oesophagus +5 mm, spinal canal +5 mm) and duplicating treatment fields with original monitor units for each CT phase. The AvIP was selected as the reference geometry for propagation as it represents the time-averaged density used for clinical dose calculation and minimizes the geometric bias inherent in selecting a single respiratory phase. Phase-specific plans were returned to Eclipse for dose calculation using the same algorithm and monitor units. Ten dose distributions, one for each phase of the 4DCT, were then re-imported into Velocity and accumulated back to the AvIP anatomy using equal temporal weighting, producing a single 4D accumulated dose distribution. See Fig. 1 for a simplified methodology. Across the cohort, this workflow generated 180 phase-specific dose distributions in addition to one average intensity projection plan per patient.

### 2.7 Endpoints

For both the airway and the airway +5 mm PRV, the following metrics were extracted from the AvIP and 4D plans: maximum point dose (D*_max_*) and near-maximum dose (*D_0.1 cm3_*). Any exceedance of SOURCE dose-volume constraints (DVCs) was recorded. Data processing was performed using Python 3.11 (Jupyter v6.5). Agreement between AvIP and 4D doses was assessed with Bland–Altman plots. Limits of agreement were set at *±*1.96× the population average DIR uncertainty. Linear regression and Pearson correlation quantified relationships between airway motion amplitude and absolute dose difference. Group comparisons by tumour location relative to the proximal bronchial tree (superior vs. inferior, anterior/posterior/lateral) and motion magnitude (*>*6 mm vs. *≤*6 mm) were performed using the two-sided Mann-Whitney *U* test; *p <* 0*.*05 was considered statistically significant.

### 2.8 Uncertainty estimation

DIR-related uncertainty was characterised per patient and dose metric by fitting a sinusoid to the ten phase doses and computing the mean absolute residual. Phase–metric values with residuals exceeding 2 standard deviations were excluded, as these were considered indicative of isolated DIR failures arising from 4DCT acquisition or phase-sorting artefacts, including slice misalignment, rather than true respiratory motion. The cohort mean of these uncertainties defined the generic error band used in Bland–Altman analysis and is reported alongside dose results to aid interpretation.

## 3 Results

The median planning target volume was 96 cm^3^ (range: 6.3–371.3), and the median superior–inferior airway displacement measured across the end-inhale and end-exhale phases was 8 mm (range: 3–15). External respiratory surrogate motion averaged 7.5 mm (range: 2–12 mm).

**Fig. 2:**
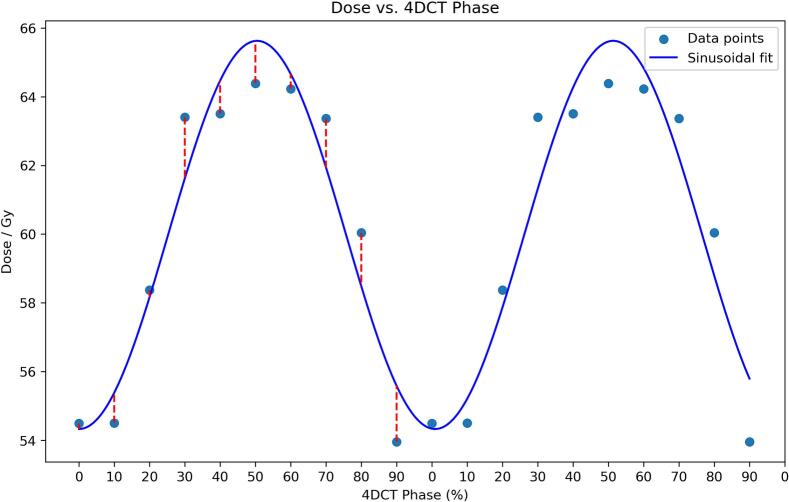
Maximum dose to Airway PRV vs. Phase of 4DCT with sinusoidal curve fit for one patient. The residuals from the curve fit were used to calculate the uncertainty of the deformable image registration.

Dose to the airway PRV (D*_max_*) exhibited a near-sinusoidal variation with respiration (see Fig. 2). Across the cohort, the mean absolute residual between phase values and the patient-specific sinusoidal fit was 0.5 Gy (airway *D_0.1 cm3_*), 0.78 Gy (airway D*_max_*), 1.1 Gy (PRV *D_0.1 cm3_*) and 1.5 Gy (PRV D*_max_*), which established the DIR-related uncertainty. Of the 720 phase-specific dose data points analysed (18 patients × 10 phases × 4 metrics), 20 individual phase–metric values (2.7%) exceeded the 2σ threshold and were excluded as outliers.

The maximum observed discrepancy in airway *D_0.1 cm3_* was 5 Gy, with an average difference of 0*.*5 *±* 1*.*2 Gy across the cohort. Similarly, for the airway PRV *D_0.1 cm3_*, 28% of patients experienced discrepancies greater than 2 Gy, and 17% had differences exceeding 3 Gy. The mean difference for airway PRV *D_0.1 cm3_* was 0*.*95 *±* 1*.*7 Gy.

**Fig. 3:**
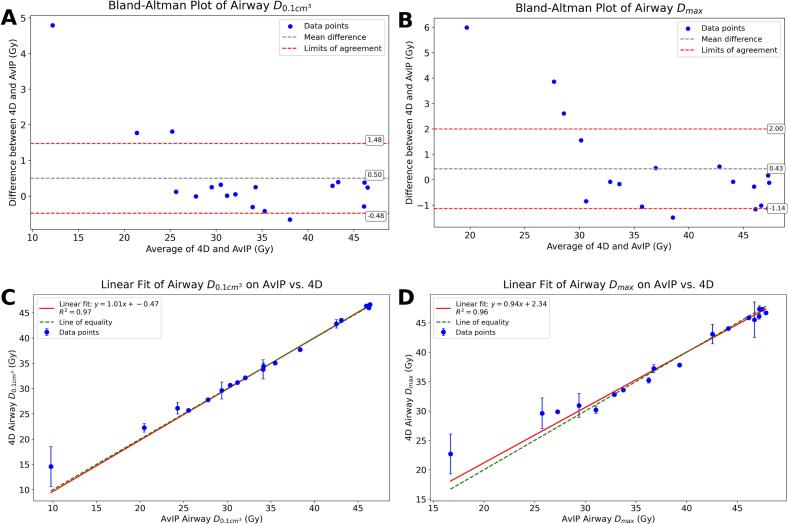
A: Bland-Altman analysis for Airway D_max_. B: Bland-Altman analysis for Airway *D_0.1 cm3_*. C: Linear regression (4D vs. AvIP) for Airway D_max_. D: Linear regression (4D vs. AvIP) for Airway *D_0.1 cm3_*.

Bland–Altman analysis demonstrated systematic underestimation of dose to the airway in AvIP-based plans when compared to 4D dose accumulation. For the airway *D_max_* and *D_0.1 cm3_* (Fig. 3A–B), the mean differences were 0.43 *±* 1*.*9 Gy and 0.5 *±* 1*.*2 Gy, respectively, with several patients exceeding the limits of agreement. The largest discrepancies (up to 6 Gy) were observed in patients with inferior tumours and airway motion exceeding 6 mm.

Linear regression plots (Fig. 3C–D) revealed strong correlations between AvIP and 4D doses (Pearson *r >* 0*.*98, *p <* 0*.*001), though clustering around dose-volume constraints (47.1 Gy and 46.7 Gy) limited interpretability.

**Fig. 4:**
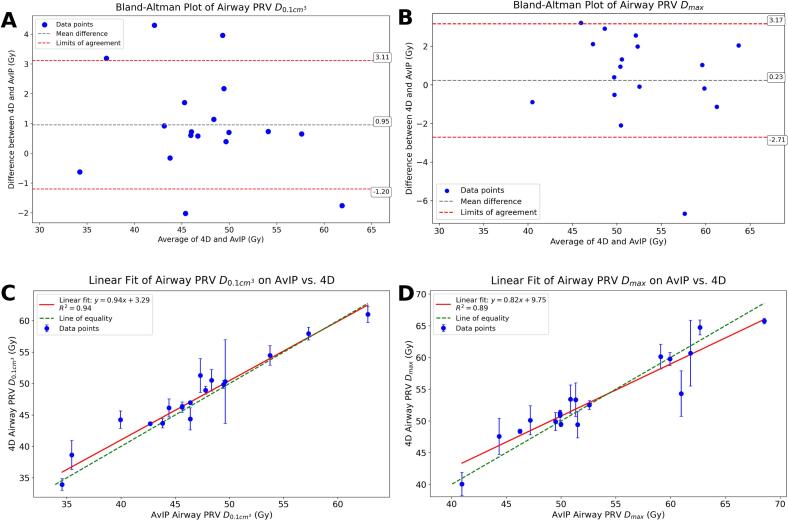
A: Bland-Altman analysis for Airway PRV D_max_. B: Bland-Altman analysis for Airway PRV *D_0.1 cm3_*. C: Linear regression (4D vs. AvIP) for Airway PRV D_max_. D: Linear regression (4D vs. AvIP) for Airway PRV *D_0.1 cm3_*.

Comparable trends were observed for the airway PRV (Fig. 4). Mean dose differences were 0.23 *±* 2*.*4 Gy for *D_max_* and 0.95 *±* 1*.*7 Gy for *D_0.1 cm3_*, with five patients exceeding *±* 3 Gy. The regression slope for PRV *D_max_* (0.79, *p <* 0*.*001) deviated further from unity, reflecting greater variation from respiratory motion.

**Fig. 5:**
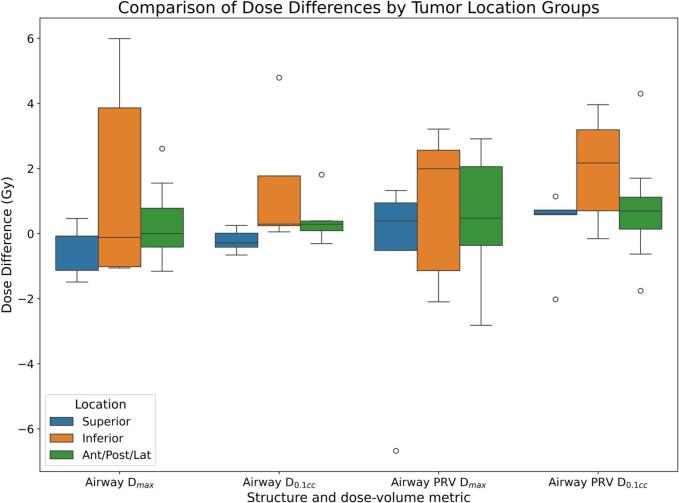
Dose difference between AvIP plan and 4D Dose accumulation (positive difference indicates higher dose on 4D plan) grouped by tumour location relative to the 1st order bronchi; the box plots show the median (central line), interquartile range (box), and whiskers extending to 1.5 times the IQR, with outliers plotted individually.

When stratified by gross tumour volume (GTV) location relative to the proximal bronchi, tumours located inferior to the first-order bronchi demonstrated a higher median PRV *D_0.1 cm3_* (+2.2 Gy; IQR 0.7–3.2 Gy) compared with superior lesions (+0.6 Gy; IQR 0.6–0.7 Gy) (Fig. 5). A difference in airway *D_0.1 cm3_* was observed between inferior and superior tumours (p = 0.03). No significant differences were observed between anterior, posterior, or lateral lesions and the reference group.

**Fig. 6:**
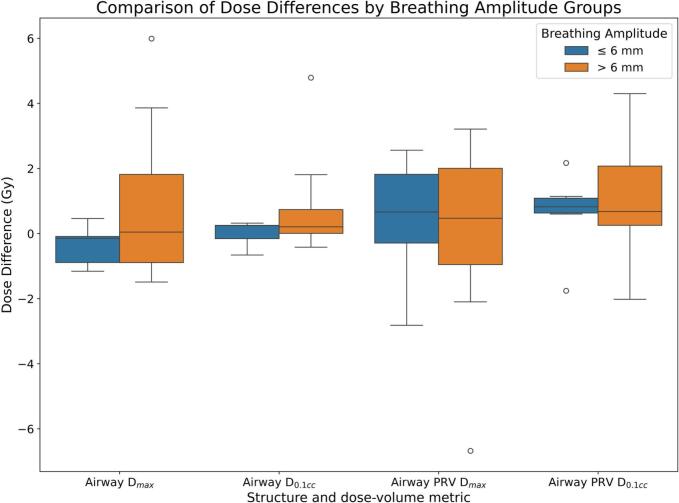
Dose difference between AvIP plan and 4D Dose accumulation (positive difference indicates higher dose on 4D plan) grouped by amplitude of breathing motion; the box plots show the median (central line), interquartile range (box), and whiskers extending to 1.5 times the IQR, with outliers plotted individually.

Grouping patients by breathing amplitude (Fig. 6) revealed that those with *>*6 mm motion (n = 9) had a greater mean increase in airway PRV *D_0.1 cm3_* than those with *≤*6 mm motion (1*.*1 *±* 1*.*9 Gy vs. 0*.*6 *±* 1*.*3 Gy).

## 4 Discussion

This study demonstrated the significant impact of intra-fraction, breathing-induced airway variations on dosimetry in central lung SABR. The results indicated that conventional AvIP-based planning and the use of a uniform 5 mm planning risk volume (PRV) around the airways [11] inadequately captured the full range of anatomical variability induced by respiratory motion. This resulted in substantial discrepancies between planned and delivered doses to critical airway structures. Five treatment plans (28%) exceeded their most critical airway DVC *only* after 4D dose accumulation. All five would have passed conventional review based solely on AvIP planning. These findings underscored that reliance on static imaging and a uniform 5 mm PRV could mask overdosing of the airways, particularly in cases with substantial cranio-caudal motion (*>*6 mm).

This work aligned with similar dose accumulation studies performed in hepatic and cardiac SABR, where breathing motion was shown to substantially alter dose distributions relative to static planning assumptions [12], [14], [15], [16]. While these studies focused on other mobile organs, the principle remains applicable: respiratory motion introduces dynamic positional variability that can undermine the accuracy of predicted dose from static or phase-averaged plans. However, unlike the liver or heart, the central airways represent a serial organ system with low tolerance to high-dose hotspots, underscoring the clinical consequences of even modest dose misestimations.

Our centre delineates OARs on the AvIP and applies a uniform 5 mm PRV. Other institutions use different workflows; some plan on the AvIP without any explicit PRV, while others contour on a single respiratory phase, most commonly the end exhale (50%) image, or on a mid-ventilation CT [17], [18], [19]. Each of these choices holds a different assumption about how respiratory motion is represented. The present study showed that these assumptions can fail even with a conservative 5 mm airway PRV, particularly in patients with large breathing motion and tumours located inferior to the main bronchi. Eliminating the PRV altogether, planning to the airway structure itself, would further increase the probability of exceeding clinically accepted DVCs once true airway motion is considered. Also, planning on a single 50% phase fixes the dose to the airway at, or near, the trough of the sinusoidal pattern observed in Fig. 2, systematically and sometimes severely under estimating the true dose. Such discrepancies approach the dose response thresholds linked to grade ≥3 airway toxicity in the SUNSET and HILUS trials [8], [10], indicating that underestimation driven by planning workflow could have clinically significant consequences.

Regardless of the planning approach an institution adopts, such margins may not reliably account for patient-specific airway motion. Incorporating either (i) internal risk volumes (IRVs) derived from measured motion or (ii) phase-resolved 4D dose accumulation into routine plan evaluation would better capture the true delivered dose to the airway [20], [21]. Personalised margins tailored to individual respiratory patterns could enhance treatment safety by improving dosimetric fidelity to anatomical reality, particularly for patients with large breathing motion or tumours located inferior to the main bronchi, where motion is more pronounced [21]. Subgroup analysis in this study further highlighted inferior tumour locations as especially vulnerable to motion-induced underestimation of dose to the airway, identifying a key patient population for more advanced, motion-aware planning strategies.

Strengths of the study included the use of deformable image registration (DIR) to derive phase-specific dose reconstructions and the incorporation of quantitative uncertainty analysis into dose comparisons. However, several limitations should be acknowledged. The retrospective design and relatively small cohort limit generalisability. The assumption that the breathing pattern during CT simulation is reliably reproduced during treatment introduces a potential temporal mismatch. Furthermore, DIR accuracy, although validated [22], [23], [24], remains a source of uncertainty, particularly in regions with low image contrast or complex anatomical deformation.

Future prospective studies with larger cohorts and real-time respiratory monitoring during treatment could further validate the observed dose discrepancies. Advances in DIR algorithms, particularly those incorporating biomechanical modelling or artificial intelligence, may also improve the accuracy and clinical utility of 4D dose reconstruction [25], [26]. The integration of personalised IRVs into clinical workflows represents a potential avenue for future work that could improve SABR planning and patient safety in the management of central and ultra-central lung tumours [20].

Ultimately, this study highlighted the dose impact of respiratory-induced airway motion in central and ultra-central lung SABR, demonstrating that conventional AvIP-based planning with a uniform PRV may underestimate dose to the airways. Such underestimations can approach clinically relevant thresholds for toxicity and may be exacerbated by planning strategies that neglect motion altogether, such as single-phase contouring. These findings support the integration of patient-specific internal risk volumes or 4D dose accumulation techniques into routine planning workflows to more accurately capture the delivered dose. Personalised, motion-incorporated approaches have the potential to improve treatment safety and better protect critical airway structures in this high-risk population.

## CRediT authorship contribution statement

**Evan Keane:** Writing – review & editing, Writing – original draft, Visualization, Methodology, Investigation, Formal analysis, Data curation. **Gerard G Hanna:** Writing – review & editing, Formal analysis. **Serena O’Keefe:** Writing – review & editing. **Pierre Thirion:** Writing – review & editing. **Ciaran Malone:** Writing – review & editing, Supervision, Conceptualization.

## Declaration of competing interest

The authors declare that they have no known competing financial interests or personal relationships that could have appeared to influence the work reported in this paper.

## References

[1] Molina J.R., Yang P., Cassivi S.D., Schild S.E., Adjei A.A. (2008). Non-small cell lung cancer: epidemiology, risk factors, treatment, and survivorship. Mayo Clin Proc.

[2] Choi J.I. (2019). Medically inoperable stage I non-small cell lung cancer: best practices and long-term outcomes. Transl Lung Cancer Res.

[3] de Ruiter J.C., van der Noort V., van Diessen J.N.A., Smit E.F., Damhuis R.A.M., Hartemink K.J. (2024). The optimal treatment for patients with stage I non-small cell lung cancer: minimally invasive lobectomy versus stereotactic ablative radiotherapy–a nationwide cohort study. Lung Cancer.

[4] Arnett A.L.H. (2019). Long-term clinical outcomes and safety profile of SBRT for centrally located NSCLC. Adv Radiat Oncol.

[5] Roach M.C. (2018). Stereotactic body radiation therapy for central early-stage NSCLC: results of a prospective phase I/II trial. J Thorac Oncol.

[6] Owen D., Sio T.T. (2020). Stereotactic body radiotherapy (SBRT) for central and ultracentral node-negative lung tumors. J Thorac Dis.

[7] Bezjak A. (2019). Safety and efficacy of a five-fraction stereotactic body radiotherapy schedule for centrally located non-small-cell lung cancer: NRG oncology/RTOG 0813 trial. J Clin Oncol.

[8] Giuliani M.E., Filion E., Faria S., Kundapur V., Vu T.T.T.T., Lok B.H. (2024). Stereotactic radiation for ultra-central non-small cell lung cancer: a safety and efficacy trial (SUNSET). Int J Radiat Oncol Biol Phys.

[9] Hoffmann L., Persson G.F., Nygård L., Nielsen T.B., Borrisova S., Gaard-Petersen F. (2022). Thorough design and pre-trial quality assurance (QA) decrease dosimetric impact of delineation and dose planning variability in the STRICTLUNG and STARLUNG trials for stereotactic body radiotherapy (SBRT) of central and ultra-central lung tumours. Radiother Oncol.

[10] Lindberg K., Grozman V., Karlsson K., Lindberg S., Lax I., Wersäll P. (2021). The HILUS-trial—A prospective nordic multicenter phase 2 study of ultracentral lung tumors treated with stereotactic body radiotherapy. J Thorac Oncol.

[11] SABR UK Consortium (2019). Stereotactic ablative body radiation therapy (SABR): a resource. R Coll Radiol.

[12] Yoganathan S.A., Maria Das K.J., Agarwal A., Kumar S. (2017). Magnitude, impact, and management of respiration-induced target motion in radiotherapy treatment: a comprehensive review. J Med Phys.

[13] Chetty I.J., Rosu-Bubulac M. (2019). Deformable registration for dose accumulation. Semin Radiat Oncol.

[14] Velec M., Moseley J.L., Eccles C.L., Craig T., Sharpe M.B., Dawson L.A. (2011). Effect of breathing motion on radiotherapy dose accumulation in the abdomen using deformable registration. Int J Radiat Oncol Biol Phys.

[15] Velec M., Moseley J.L., Craig T., Dawson L.A., Brock K.K. (2012). Accumulated dose in liver stereotactic body radiotherapy: positioning, breathing, and deformation effects. Int J Radiat Oncol Biol Phys.

[16] Johnson-Hart C., Price G., McWilliam A., Green A., Faivre-Finn C., Van Herk M. (2020). Impact of small residual setup errors after image guidance on heart dose and survival in non-small cell lung cancer treated with curative-intent radiotherapy. Radiother Oncol.

[17] Thomas S.J. (2019). An evaluation of the mid-ventilation method for the planning of stereotactic lung plans. Radiother Oncol.

[18] Cooper D. (2024). Motion-inclusive treatment planning to assess normal tissue dose for central lung stereotactic body radiation therapy. Pract Radiat Oncol.

[19] Wolthaus J.W.H. (2006). Mid-ventilation CT scan construction from four-dimensional respiration-correlated CT scans for radiotherapy planning of lung cancer patients. Int J Radiat Oncol Biol Phys.

[20] Nardone V. (2020). 4D CT analysis of organs at risk (OARs) in stereotactic radiotherapy. Radiother Oncol.

[21] Brunner T.B. (2016). Simultaneous integrated protection: a new concept for high-precision radiation therapy. Strahlenther Onkol.

[22] Hardcastle N., Van Elmpt W., De Ruysscher D., Bzdusek K., Tomé W.A. (2013). Accuracy of deformable image registration for contour propagation in adaptive lung radiotherapy. Radiat Oncol.

[23] Kadoya N., Fujita Y., Katsuta Y., Dobashi S., Takeda K., Kishi K. (2014). Evaluation of various deformable image registration algorithms for thoracic images. J Radiat Res.

[24] Hoffmann C., Krause S., Stoiber E.M., Mohr A., Rieken S., Schramm O. (2014). Accuracy quantification of a deformable image registration tool applied in a clinical setting. J Appl Clin Med Phys.

[25] Samavati N., Velec M., Brock K.K. (2016). Effect of deformable registration uncertainty on lung SBRT dose accumulation. Med Phys.

[26] Xiao H., Ren G., Cai J. (2020). A review on 3D deformable image registration and its application in dose warping. Radiat Med Prot.

