# Supplementary material for: Impact of respiratory motion on dose to the airways in central and ultra-central lung stereotactic ablative body radiotherapy
Source: Phys Imaging Radiat Oncol. 2026 Feb 26;38:100937. doi: 10.1016/j.phro.2026.100937 (PMC12969133; doi:10.1016/j.phro.2026.100937)
Supplement: Supplementary Data 1 [file mmc1.pdf]

Table 1. Target Dose Coverage Constraints

Structure	Optimum	Deviation
**PTV**	D95%=100%	D95%≥75%
	D99%≥90%	–
	PTV Dmax:Central: 110–120%Peripheral: 110–140%	All sites: 100–110%Central: 120–125%
**GTV**	D99%≥100%	D99%≥75%

Table 2. Central OAR constraints

Structure	Volume (cm³)	Optimum (Gy)	Acceptable Deviation (Gy)
**Airway**	0.1	32	44
	0.03	–	47.1

Table 3. SOURCE Trial / Ultra-central OAR Constraints (8 Fractions)

Structure	Volume (cm³)	Eligibility Criteria (Gy)	Acceptable Deviation (Gy)
**Airway**	0.1	44	46.68
	0.03	47.1	50
